# Left hemispheric deficit in the sustained neuromagnetic response to periodic click trains in children with ASD

**DOI:** 10.1186/s13229-020-00408-4

**Published:** 2020-12-31

**Authors:** T. A. Stroganova, K. S. Komarov, O. V. Sysoeva, D. E. Goiaeva, T. S. Obukhova, T. M. Ovsiannikova, A. O. Prokofyev, E. V. Orekhova

**Affiliations:** 1 Center for Neurocognitive Research (MEG Center), Moscow State University of Psychology and Education, Moscow, Russian Federation; 2 MedTech West and the Institute of Neuroscience and Physiology, Sahlgrenska Academy, The University of Gothenburg, Gothenburg, Sweden; 3 Institute of Higher Nervous Activity, Russian Academy of Science, Moscow, Russian Federation

**Keywords:** Autism spectrum disorders (ASD), Magnetoencephalogram (MEG), Pitch processing, 40 Hz clicks, Auditory steady state response (ASSR), Sustained field (SF), Children

## Abstract

**Background:**

Deficits in perception and production of vocal pitch are often observed in people with autism spectrum disorder (ASD), but the neural basis of these deficits is unknown. In magnetoencephalogram (MEG), spectrally complex periodic sounds trigger two continuous neural responses—the auditory steady state response (ASSR) and the sustained field (SF). It has been shown that the SF in neurotypical individuals is associated with low-level analysis of pitch in the ‘pitch processing center’ of the Heschl’s gyrus. Therefore, alternations in this auditory response may reflect atypical processing of vocal pitch. The SF, however, has never been studied in people with ASD.

**Methods:**

We used MEG and individual brain models to investigate the ASSR and SF evoked by monaural 40 Hz click trains in boys with ASD (*N* = 35) and neurotypical (NT) boys (*N* = 35) aged 7–12-years.

**Results:**

In agreement with the previous research in adults, the cortical sources of the SF in children were located in the left and right Heschl’s gyri, anterolateral to those of the ASSR. In both groups, the SF and ASSR dominated in the right hemisphere and were higher in the hemisphere contralateral to the stimulated ear. The ASSR increased with age in both NT and ASD children and did not differ between the groups. The SF amplitude did not significantly change between the ages of 7 and 12 years. It was moderately attenuated in both hemispheres and was markedly delayed and displaced in the left hemisphere in boys with ASD. The SF delay in participants with ASD was present irrespective of their intelligence level and severity of autism symptoms.

**Limitations:**

We did not test the language abilities of our participants. Therefore, the link between SF and processing of vocal pitch in children with ASD remains speculative.

**Conclusion:**

Children with ASD demonstrate atypical processing of spectrally complex periodic sound at the level of the core auditory cortex of the left-hemisphere. The observed neural deficit may contribute to speech perception difficulties experienced by children with ASD, including their poor perception and production of linguistic prosody.

## Background

Autism spectrum disorder (ASD) is a neurodevelopmental disorder, characterized by challenges in social interaction, deficient verbal and non-verbal communication skills, restricted interests, repetitive behaviors, and is often associated with low intelligence [1]. Although atypical development of spoken language is among the core symptoms of ASD, language impairments are highly variable across individuals with ASD, ranging from lack of spoken language to incomplete understanding of language pragmatics [2]. The neural origin of spoken language deficiency in people with ASD remains poorly understood.

Neuroimaging studies have shown that atypical development in children with ASD may originate from an early aberration of the temporal cortex maturation, which is specific to the left hemisphere [3]. It remains unknown, however, when and where in the brain a left-hemispheric speech processing deficit arises in individuals with ASD. One of the most intriguing aspects of this problem is whether such a lateralized deficit exists already at the low level of cortical hierarchy—in the regions of the auditory core confined by Heschl’s gyrus [4] that extracts the periodicity/pitch from the acoustic input. This ‘low-level’ neurofunctional deficit may manifest itself in several ways. First, it might result in atypical frequency encoding of the auditory input that relies on tonotopic neuronal representations of distinct frequency channels, inherited by the primary auditory cortex (A1) from subcortical auditory pathways [5]. Second, it may cause abnormal processing of the temporal regularities in the amplitude envelope of a spectrally complex auditory signal. While slow temporal modulations at frequencies from 4 to 16 Hz play an important role in speech comprehension [6, 7], those of higher frequencies (> ~ 30 Hz) are essential for perception of vocal pitch [8]. Unlike pure tones, periodic temporal modulation of the complex sound (e.g. repetitive mixture of harmonics or repetitive transients/noise) is not solely place-coded in the A1; it is also processed in another region of the core auditory cortex [9]. Neuronal findings in monkeys [10, 11] and functional neuroimaging in humans [12] have shown that the so-called ‘pitch processing center’ in the auditory cortical hierarchy is localized downstream to A1 in the more anterolateral portion of Heschl’s gyrus, at the border of the core and the belt auditory cortical areas. Given that individuals with ASD, regardless of their intelligence quotient (IQ) and general language skills, have abnormalities in perception of pitch in speech signals, as well as difficulties with production of adequately intonated speech [13, 14], a putative low-level neural deficit in encoding temporal regularity in this clinical group merits careful investigation.

While many studies associate pitch processing primarily with the right auditory cortex [15], there is strong evidence that temporal regularities perceived as a pitch are processed bilaterally, but differently in the left and right hemispheres [16, 17]. The low-level processing of temporal regularities in spectrally complex sound can be investigated by measuring lateralized neural auditory responses to sequences of periodic clicks presented monaurally to the left and right ears. According to findings in healthy adults, the prolonged periodic click trains evoke two types of magnetoencephalogram (MEG) responses in the auditory cortex—the oscillatory auditory steady-state response (ASSR) at the frequency of stimulation and the sustained deflection of the magnetic field (‘sustained field’ [SF]) [18–21].

Although both the SF and ASSR had been previously associated with processing of periodicity/temporal regularity [19, 21], only the ASSR has been examined in individuals with ASD [22–25]. The ASSR is an oscillatory response phase-locked to the onset of each single auditory event, i.e. each click in the train. The maximal ASSR is observed at the stimulation frequency of 40 Hz that is thought to represent the resonance frequency of the A1 neural circuitry [26]. The interest of researchers to the 40 Hz ASSR in people with ASD has been driven by its putative link to local connectivity disturbances in these disorders [25] and numerous reports on its robust reduction in patients with schizophrenia (for a review see [27]).

In contrast to schizophrenia research, only a few studies have examined the 40 Hz ASSR in ASD, and the results of these studies are inconsistent. Researchers have reported a reduction in the ASSR either bilaterally in adolescents and adults with ASD [24], or unilaterally in the left hemisphere, both in children with ASD and their first-degree relatives ([25] and [28], respectively). Of note, Seymour et al. [24] found reduction of the ASSR in adolescents with ASD only in the late interval of the stimulation (> 500 ms), while the studies that reported the ASD-related ASSR differences in children and adults, observed them already in the 200–500 ms interval [25, 28]. In addition to uncertainty regarding lateralization and timing of the ASSR abnormality, two recent studies that similarly to Seymour et al. applied long (1 s) stimulation intervals, found the ASSR to be fairly normal in both hemispheres of children with ASD [22, 29]. Edgar and colleagues also investigated the developmental trajectory of the ASSR and suggested that the discrepancy with the previous results was due to relatively late maturation of this response in humans. They observed that the ASSR was typically weak and unreliable before puberty, which might preclude detection of its potential abnormalities in children with ASD. The reasons for the conflicting findings may include age of the participants [30], high heterogeneity of the ASD population, as well as the type of stimulation (monaural vs binaural) used in different studies. Monaural compared with binaural stimulation is known to produce larger hemispheric asymmetry [31] and, therefore, is more suitable to reveal putative hemisphere-specific differences in ASSR in people with ASD.

Although the ASSR reflects processing of stimulus periodicity within a certain range of frequencies, it is hardly relevant for pitch processing in the auditory cortex. Indeed, the ASSR originates from A1 and gradually decreases and almost disappears at frequencies of 75–150 Hz [32]—the fundamental frequencies of the human voice that mainly determine what is perceived as the pitch of the voice [33]. The higher-order processing associated with perception of pitch is rather reflected in another component—the SF generated in the anterolateral region of Heschl’s gyrus [19, 20, 34]. The SF is a slowly developing direct current (DC) shift with negative polarity (in the electroencephalogram [EEG]) that is locked to the onset of temporally modulated auditory stimulation and continues throughout its duration [35]. Apart from click trains, the SF can be triggered by any periodic spectrally complex sounds, including amplitude-modulated tones and speech sounds, and is very sensitive to periodicity, which is perceived as a sustained pitch [18, 34]. The ‘pitch processing center’ that generates the SF does not belong to the primary auditory cortex, despite the anatomical proximity and cytoarchitectonic similarity of these two cortical regions [4]. Based on these findings, it has been suggested that the SF reflects the integration of pitch information across the frequency channels of the primary auditory cortex, where the sound frequencies are represented as a tonotopic frequency map [19].

Given the relevance of the SF to pitch processing, it is surprising that this neural response escaped the attention of researchers who studied the 40-Hz ASSR in ASD. Moreover, in neurotypical (NT) children, the SF response to the 40 Hz clicks has only been described in one study (mean age of participants was 12 years) but its characteristics (localization, hemispheric asymmetry) have not been analyzed [36]. Therefore, it is not known whether the properties of the SF responses to the 40-Hz clicks in children and adults are similar. Taking into account the protracted maturation of the auditory evoked responses [37–40], the properties of the SF might change with age. If child and adult SF are homologous, studying this response in children with ASD may reveal the suspected abnormalities in processing of pitch in the ‘pitch processing center’. Moreover, exploration of the SF hemispheric asymmetry using a monaural presentation of periodic clicks may shed light on the hemisphere-specific deficit in pitch processing in ASD.

In this study we investigated both types of auditory evoked fields (AEF) associated with processing of sound periodicity in children with ASD, namely the ASSR and SF. For a more reliable analysis of hemispheric asymmetries, we presented 40 Hz click trains monaurally to the left and right ears. We recorded auditory responses using MEG and applied individual head models for source analysis. This made it possible to localize the cortical sources of both components and to determine the direction of the source current, which is important for the neurophysiological interpretation of the results. Given the lack of knowledge about the properties of the SF in children, we *first*, compared the spatial and temporal characteristics of this auditory response in NT children and adults. *Second*, we compared the ASSR and SF in NT children and those with ASD. We expected that if there is a difference between the groups, it might be specific to a particular type of response and/or confined to one hemisphere. *Third,* we examined whether inter-individual variations of the ASSR and SF—the components associated with low-level processing of non-speech periodic sound—correlate with cognitive deficit and the core symptoms of autism in children with ASD.

## Methods

### Participants

The study included 35 boys with ASD aged 7–12 years and 35 NT boys of the same age range. The participants with ASD were recruited from rehabilitation centers affiliated with the Moscow University of Psychology and Education. The ASD diagnosis was confirmed by an experienced psychiatrist and was based on the *Diagnostic and Statistical Manual of Mental Disorder* (5th ed.) criteria as well as an interview with the parents/caregivers. In addition, parents of all children were asked to complete the Russian translation of three parental questionnaires: Social Responsiveness Scale for children [41], Autism Spectrum Quotient (AQ) for children [42] and Social Communication Questionnaire (SCQ-Lifetime) [43]. None of the NT participants had known neurological or psychiatric disorders. All participants had normal hearing according to medical records. In children, IQ has been evaluated through standard scores on K-ABC subscales (Simultaneous and Sequential), as well as by calculating the Mental Processing Index (MPI) [44]. Table 1 presents characteristics of the pediatric samples. To compare child and adult auditory responses to click trains, we included in the study 10 NT adults (ages 22–36 years, mean ± standard deviation [SD] = 28.5 ± 4.1, 5 females).

**Table 1:** Characteristics of the pediatric samples (mean, standard deviation [SD], range and group differences)

	ASD	NT	*p*	T
Age (years) *N*_NT/ASD_ = 35/35	9.69 ± 1.5 (7.2–12.3)	10.08 ± 1.5 (7.3–12.9)	0.29	1.09
Sequential IQ *N*_NT/ASD_ = 35/32	78.9 ± 19.1 (49–127)	106.8 ± 13.7 (77–134)	< 1e−6	6.93
Simultaneous IQ *N*_NT/ASD_ = 35/32	89.2 ± 22.0 (43–134)	123.3 ± 15.3 (91–151)	< 1e−6	7.43
MPI Standard IQ *N*_NT/ASD_ = 35/32	84.3 ± 22.0 (45–128)	121.2 ± 12.6 (94–145)	< 1e−6	8.51
SRS *N*_NT/ASD_ = 34/32	104.8 ± 23.9 (57–157)	45.2 ± 22.6 (3–91)	< 1e−6	− 10.4
SCQ-life *N*_NT/ASD_ = 33/32	24.7 ± 5.4 (11–34)	6.6 ± 4.2 (0–19)	< 1e−6	− 15.0
AQ *N*_NT/ASD_ = 33/32	90.3 ± 12.2 (61–118)	53.7 ± 13.0 (23–76)	< 1e−6	− 11.7

‘N_NT/ASD_’ indicates the number of participants in the corresponding groups for whom data were available

The Ethical Committee of the Moscow University of Psychology and Education approved this investigation. All participants and/or their caregivers provided their verbal assent to participate in the study and were informed about their right to withdraw from the study at any time during testing. The adult participants and guardians of all children gave written informed consent after the experimental procedures had been fully explained.

### Auditory stimuli

The stimuli—500 ms trains of 40 Hz clicks—were delivered via plastic ear tubes inserted in the ear channels. The intensity level was set at 60 dB sound pressure level (SPL). The duration of each click was 2 ms and the stimulus onset asynchrony was 25 ms, resulting in a train of 20 clicks. Intervals between the trains were fixed at 1000 ms. The stimuli were organized in two blocks, each containing 100 click trains presented to one ear. The order of the ‘left’ and ‘right’ blocks was counterbalanced between subjects. The experiment lasted for approximately 5 min. Participants were instructed to ignore the auditory stimulation and watched a silent video of their choice during the experiment.

**Fig. 1:**
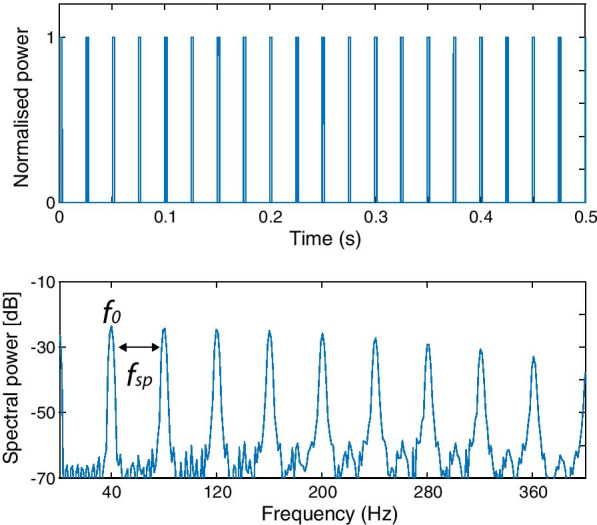
Waveform (upper panel) and power spectrum (lower panel) of the 40 Hz click train. Periodicity (‘pitch’) can be analyzed by the auditory system based either on the fundamental frequency (*f*_0_) or on the common devisor of the sound’s harmonics (*f*_sp_)

Waveform and spectral composition of the stimulus are presented in Fig. 1. The signal energy is concentrated at 40 Hz (*f*_0_, fundamental frequency), as well as at the higher frequency harmonics of comparable physical intensity. Periodicity (‘pitch’) in such spectrally complex sound can be analyzed by the auditory system based either on the lowest harmonic present (*f*_0_), or on the highest common devisor of the sound’s harmonics (*f*_sp_) [16, 45].

### MEG acquisition and preprocessing

MEG was recorded in a sitting position in a magnetically-shielded room using a 306-channel MEG scanner (Vectorview, Elekta-Neuromag) that comprises 204 orthogonal planar gradiometers and 102 magnetometers in 102 locations above the participant’s head. An electrooculogram (EOG) was recorded using four electrodes placed at the outer canthi of the eyes and above and below the left eye. To monitor heartbeats, one electrocardiogram (ECG) electrode was placed at the manubrium sterni and the other one at the mid-axillary line (V6 ECG lead). The signal was sampled at 1000 Hz and filtered on-line with a bandpass filter of 0.1–330 Hz.

Prior to the MEG recording, the positions of HPI coils, fiducial points and additional points on the head and face were digitized using the 3D digitizer ‘FASTRAK’ (Polhemus, Colchester, VT, United States). The subject’s head position inside the MEG helmet was assessed every 4 ms. The temporal signal space separation (tSSS) with correlation limit 0.9 and movement compensation implemented in MaxFilter software (Elekta-Neuromag) were used to suppress environmental noises and compensate for head movements. Maximal displacement of the head origin during MEG registration was < 21.5 mm and did not differ between the ASD and NT groups of children (*T* test: *p* = 0.15). The data were converted to standard head position (*x* = 0 mm; *y* = 0 mm; *z* = 45 mm).

We used only gradiometers for analysis. MEG data preprocessing was performed using MNE-python software [46] and custom python scripts. The raw data were notch-filtered at 50, 100 and 150 Hz using MNI toolbox (v0.19) default options (stop band of the notch filter = frequency/200 Hz). To detect the sustained component of the auditory response we have chosen not to apply an off-line high-pass filter. We used independent component analysis (ICA) performed on the continuous data to reject components associated with eye movements, heartbeats and muscle artifacts. The data segments characterized by too low (< 1e−13 T/m) or too high (> 4000e−13 T/m) amplitudes were excluded from ICA and the following analysis. We used a semi-automated approach implemented in the MNE-python for detection and removal of electrocardiographic (ECG) and vertical electrooculographic (EOG) artefacts. To ensure that the automatically detected ICA components (up to 3 for ECG and up to 2 for EOG) were caused by the artefacts, we checked their timecourses and spatial distributions. Besides, in some children, up to two EMG components, characterized by spiky timecourses and flat spectra, were visually identified and also removed. The number of rejected components per subject did not differ in the NT and ASD groups (NT: mean = 3.8, SD = 0.96; ASD: mean = 3.9, SD = 1.22, *p* = 0.7). The cleaned signal was epoched from − 500 to + 1000 ms relative to the click train onset. We then rejected the epochs contaminated by occasional SQUID ‘jumps’, based on thresholding the amplitude of the 100 Hz high-passed signal at 3 SD from the mean (the approach used in Fieldtrip [47]). The trials were then visually inspected to remove the remaining epochs contaminated by bursts of muscle activity. The minimal number of the clean epochs per subject and condition was 53 (range 53–97, see Additional file 7: Figure S1). The mean number of epochs was 89/89 (left/right ear stimulation) in adults, 86/88 in NT children and 77/77 in children with ASD.

### Source localization

To obtain individual source models we used T1-weighted magnetic resonance imaging (MRI) recordings acquired on a 1.5 T Toshiba ExcelArt Vantage scanner (repetition time [TR] = 12 ms, echo time [TE] = 5 ms, flip angle = 20°, slice thickness = 1.0 mm, voxel size = 1.0 × 1.0 × 1.0 mm^3^). Cortical reconstruction and volumetric segmentation were performed with the Freesurfer image analysis suite [48]. The gray-matter segment was used to construct a continuous triangular high-density mesh representing the neocortex. The following analysis was performed using the Brainstorm software [49]. The MEG–MRI co-registration was performed using six reference points (nasion, left and right preauricular points, anterior commissure, posterior commissure and interhemispheric cleft) and the additional points. For every subject the Freesurfer cortical meshes were downsampled to have 15,000 vertices. To compute brain models, we implemented the Brainstorm method ‘overlapping spheres’, which fits one local sphere for each sensor [50]. Source reconstruction was performed using the standardized low-resolution brain electromagnetic tomography (sLORETA) [51]. Noise covariance was estimated in the − 500 to 0 ms time interval preceding the stimulation onset. To facilitate comparison between subjects, the individual sLORETA results were morphed to the ‘Colin 27’ template brain provided by Brainstorm.

### Anatomical labels

Sources of the 40 Hz ASSR and SF were estimated within the left and the right auditory cortex. Based on the previous studies that used periodic auditory stimuli [19, 20, 52] we have limited the search for cortical sources by the anatomical labels that overlapped or adjoined the left and right auditory cortices: planum temporale of the superior temporal gyrus, transverse temporal sulcus, transverse temporal gyrus of Heschl, planum polare of the superior temporal gyrus, inferior segment of the circular sulcus of the insula, lateral superior temporal gyrus and posterior ramus of the lateral sulcus ('Destrieux' cortical atlas [53]).

### The 40 Hz ASSR

For the ASSR analyses, we applied the inverse operator to single epochs filtered between 20 and 70 Hz using band-pass Butterworth IIR filter implemented in Fieldtrip (version 2016-12-31, with default parameters). We then calculated 40 Hz inter-trial phase coherence (ITPC)—a measure of phase consistency over trials*,* with ITPC = 1 reflecting maximal phase consistency across trials and ITPC = 0 indicating maximal phase variability across trials. Researchers have shown that ITPC is a more reliable measure compared with the total power in the same frequency range [54], and it is also more sensitive to developmental increase in the 40 Hz ASSR [29]. To calculate ITPC, we performed time–frequency analysis using multitapers with 200 ms sliding time window moving with 0.01 s steps. The frequency smoothing parameter (‘tapsmofrq’) was set to 4. For each subject and stimulation condition the ITPC values were averaged in the 38–42 Hz frequency range and in the interval between 200 and 500 ms after the click train onset (further called ‘40 Hz ITPC’). Previous studies have shown that the above time interval corresponds to the ‘steady-state’ phase of the ASSR [55]. To estimate the NT vs ASD differences, for each child we calculated the 40 Hz ITPC averaged over 30 vertices, which had the highest ITPC values in the combined group of children and were considered as a common cortical region of interest (ROI) for the ASD and NT groups. To estimate random 40 Hz ITPC values, we computed the average baseline ITPC in the interval from − 400 to − 100 ms prior to the stimulation onset in the same ROI. To eliminate the effect of statistical bias associated with between-group differences in trial numbers, for statistical analysis we used baseline-normalized ITPC z-values [56], which are further referred to as ‘z-ITPC’. In addition to ITPC, we computed the ASSR power as a percent power change within 200–500 ms post-onset interval relative to baseline ([stimulation − baseline]/baseline × 100%). To take into account interindividual anatomical variability, we also estimated the ASSR z-ITPC and power in the individually defined 30 vertices with maximal values of the respective parameters (‘maximal vertices’).

To determine cortical localization of the ASSR, for each participant we estimated MNI coordinates of the vertices with the maximal 40 Hz ITPC values in the left and right hemispheres, contralateral to the stimulated ear. For this analysis we excluded subjects who had 40 Hz ITPC values lower than 0.18, which was the maximal baseline value observed across subjects and conditions. Individual coordinates were then used to explore the differences in cortical localizations of the ASSR between the ASD and NT groups, as well as between the ASSR and SF in each experimental group.

### The SF

**Fig. 2:**
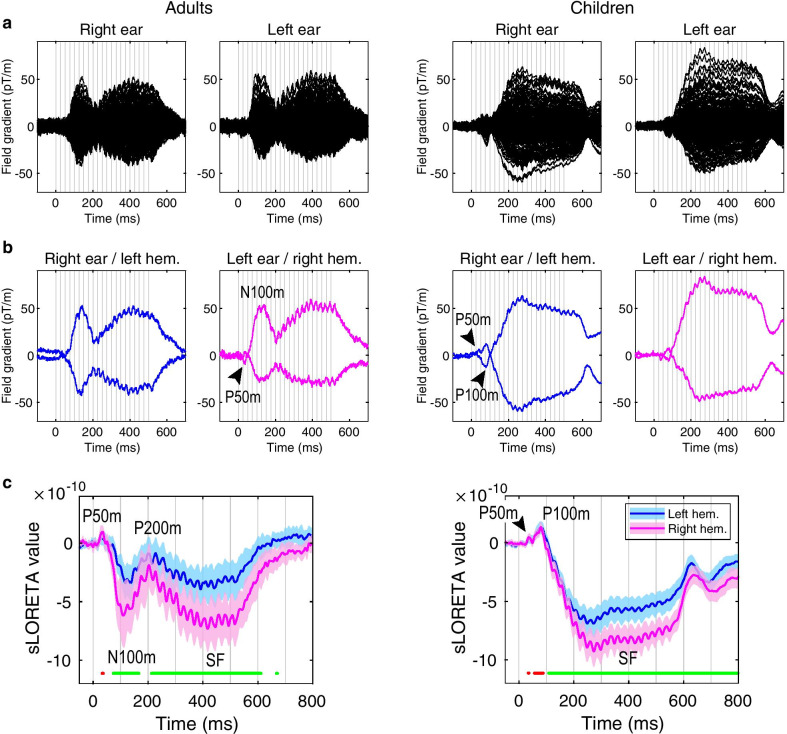
Transient and sustained auditory responses to 40 Hz click trains in neurotypical adults and children: grand average plots. Here and hereafter, zero time corresponds to the click train onset. The auditory steady-state response (ASSR) is present in all plots as an oscillatory 40 Hz response, which overlaps with the transient components and the sustained field (SF). **a** ‘Butterfly’ plot of 204 gradiometers for the right- and left-ear stimulation conditions. Vertical gray lines mark onsets of each click in the train. **b** Auditory responses in the selected left (for the right ear stimulation) and right (for the left ear stimulation) gradiometers. The selected gradiometers represent the channels measuring maxima of the outgoing/‘positive’ and incoming/ ‘negative’ spatial derivatives of magnetic field. The signal deflections corresponding to the P50m and P100m (the latter is present only in children) are marked by arrows. **c** Averaged timecourses of the source current at the SF group maxima. The shaded area marks the 95% confidence interval of the mean. Dots under the curves correspond to significant positive (red) and negative (green) deflections of the source current in the right hemisphere (*p* < 0.01, false discovery rate corrected). Note that direction of the SF source current is negative in both age groups despite marked differences in the preceding transient evoked components

The signal was first averaged over epochs and the individual timecourses were obtained for each vertex source and were baseline-corrected using the pre-stimulus baseline from − 200 to 0 ms prior to click train onset. To find MNI coordinates of the left and right SF maxima, we calculated average absolute current amplitude in the 200–500 ms interval. We chose this time interval based on results from a previous study in adults [21]; this range also included the SF maxima in both our adult and children groups (Fig. 2). We calculated the groups’ average SF coordinates (NT adults, NT children, ASD children) by averaging the individual SF coordinates across participants.

To define the SF ROI for the group analysis of the SF amplitude, we averaged absolute amplitudes in the 200–500 ms interval for all children (NT + ASD) or adults and found 30 vertices with the greatest resulting values. We then calculated the individual SF timecourses as an average across these 30 ‘common maximal vertices’. Given that the shift in polarity indexes a distinct neural response, to calculate the average SF timecourses we retained the sign of the activation [57]. Visual inspection of the results revealed that the direction of the SF current in the auditory cortex had predominantly negative polarity (for videos of the full timecourses in all groups and in both contralateral hemispheres see Additional files 1–6). Being similar in the waveform, in some vertices the SF could have an opposite—positive—polarity. A change in the polarity occurs when the patches of neural activation spread to the opposite walls of a sulcus/gyrus. Because we defined the SF ROI based on the absolute amplitude of the signal morphed to the template brain, its location has not been affected by polarity mismatches. However, to avoid cancellation of the signed timecourses, one should consider the polarity mismatches before averaging (e.g. [58]). The number and coordinates of the vertex sources demonstrating occasional polarity mismatches within the ROI varied among subjects because of individual differences in cortical morphology. Indeed, Heschl’s gyrus exhibits a highly variable morphology that includes one to three gyri per hemisphere, with the number of gyri varying between hemispheres [59, 60]. For each subject, we therefore flipped polarity of the signal from those sources where the current timecourse in the stimulation interval (0–500 ms) was anti-correlated (*R* < 0) with the ROI grand average timecourse. As the SF source current had a predominantly negative sign, this resulted in occasionally flipping positive-polarity timecourses before the averaging. The number of ‘flips’ did not differ in children with ASD and NT children (left hemisphere: *t* = − 0.05, *p* = 0.95; right hemisphere: *t* = 0.26, *p* = 0.79).

For each subject, the averaged signal was low-passed at 9 Hz (fir filter, filter order 300 points) to filter out the 40 Hz ASSR and other high-frequency activity. We used Matlab function ‘filtfilt’ that provides reverse filtering with zero-phase distortion. The SF—the component characterized by sustained current with negative polarity—had, on average, an earlier onset in children than in adults (Fig. 2c). Therefore, for the repeated measures analysis of variance (rmANOVA) analysis of the SF timecourses in children, we set the lower boundary of the SF time interval to 150 ms and then calculated the averaged SF amplitude at four consecutive 100 ms intervals after the click train onset: 150–250, 250–350, 350–450 and 450–550 ms. Further, we complemented this analysis by examining the group difference in the whole SF timecourse on a point-by-point basis to characterize more precisely the timing and duration of the group differences in the SF.

To ensure that the group differences in the SF timecourse are not caused by selection of a common ROI, for each subject we also analyzed the SF parameters in the individually defined 30 maximal vertices. This procedure considered interindividual anatomical variability and slight inaccuracies in MEG–MRI co-registration that might vary between subjects.

### Statistical analysis

We used STATISTICA software (TIBCO Software Inc, CA, United States) for statistical analysis. For the nearly normally distributed SF amplitudes, we used rmANOVA to test for the effects of the ear of the stimulation (left, right), hemisphere (contralateral, ipsilateral), time (four time intervals after the stimulation onset) and their interactions with the group factor (ASD vs TD). Violations of the sphericity assumption of the rmANOVA were corrected by adjusting the degrees of freedom with the Greenhouse–Geisser correction method. We used Bonferroni-corrected planned comparisons to evaluate the between-group differences in the SF amplitude averaged across each of the four successive 100 ms time intervals in both contralateral hemispheres. The alpha level was set at 0.006 for a single pairwise comparison (0.05/8). For abnormally distributed ASSR parameters, we used the nonparametric tests to compare the ITPC and power between groups (Mann–Whitney *U* test) and hemispheres (Wilcoxon matched pairs test). To ensure that the presence/absence of group differences in the ASSR or in the SF parameters were not due to a mismatch between a common ROI and individual activation, we repeated group analyzes twice: for the common ROI and for the individually defined ROI (30 ‘maximal vertices’; see above).

To compare SF and ASSR timecourses between the ASD and NT children on a point-by-point basis, we used the Mann–Whitney *U* test. To correct for multiple comparisons we pooled the data points for contra- and ipsilateral hemispheres and for left and right ear stimulation conditions and applied false discovery rate (FDR) procedure separately for the SF amplitude (for 500 × 4 values in 0–500 ms interval), z-ITPC (30 × 4 values in 200–500 ms interval) and ASSR power (30 × 4 values in 200–500 ms interval).

To examine correlation between neural responses and psychological variables, we calculated Pearson correlations for approximately normally distributed SF source amplitudes and Spearman correlations for ASSR parameters whose distribution deviated from normal in at least one of the experimental groups. We also reported both uncorrected and Bonferroni-corrected *p* values.

## Results

### Psychometric results

*IQ.* Children with ASD had significantly lower IQ than NT children (Table 1). Variability in MPI scores was high in the ASD sample and ranged from ‘very low’ to ‘higher than the average’ scores on all three scales.

*Autism scores*. According to each of the three parents’ questionnaires used, the severity of autism symptoms was significantly higher in the ASD compared with the NT sample (Table 1). There were high correlations between all three ‘autism severity’ scales in both the ASD (Pearson correlation coefficients for SRS/SCQ-life: *R* = 0.53, *p* = 0.003; SRS/AQ: *R* = 0.52, *p* = 0.003; SCQ-life/AQ, *R* = 0.58, *p* = 0.001) and the NT (Pearson correlation coefficients for SRS/SCQ-life: *R* = 0.60, *p* < 0.001; SRS/AQ, 0.84, *p* < 0.001; SCQ-life/AQ, 0.51, *p* = 0.003) participants. To construct a unified ‘Autism Score’ for neuro-behavioral correlation analysis, we reduced dimensionality of the data by extracting the common variance shared by the three questionnaires. We only used data from children with ASD to construct the ‘Autism Score’. The values obtained from the three autism scales were z-transformed before performing the principle component analysis (PCA). In the subjects with missing data on some of the scales (*N* = 4), we substituted the missing values with the mean of the available scales. The individual factor scores on the first principle component, which accounted for 71% of the variance in the ASD group, were then used as the integrative ‘Autism Scores’. Children with positive Autism Scores scored higher than the average for the ASD group on autism severity, while negative Autism Scores indicated relatively milder autism symptoms. There was a negative correlation between the ‘Autism Score’ and IQ (MPI Standard) (Pearson correlation coefficient *R*_(31)_ = − 0.36, *p* = 0.049).

### The auditory response waveforms in children and adults

To investigate whether the SF is present in 7–12-year-old children and whether it is homologous to the ‘adult’ SF, we compared the entire waveform of the auditory evoked response to click trains in the NT children and adults at the sensor level and at the level of cortical sources. To visualize all the main components of the auditory response—the transient components, the ASSR and SF—we set the low-pass filter at 100 Hz.

Figure 2 displays the grand average sensor-space plots (A, B) and sLORETA timecourses in the SF group maxima within the left and right hemispheres (C; see Methods for details). There were marked differences in the auditory responses to click trains between the NT children and adults.

In adults, the stimulation onset evoked a sequence of transient obligatory MEG responses: a small but distinguishable P50m at around 40 ms, followed by a much more prominent N100m of the opposite polarity peaking at around 115 ms after the stimulation onset. The strength of the negative current decreased at 200 ms due to an evolving ‘positive’ P200m. These transient components overlapped with a slowly developing shift of magnetic field (SF), which had the same polarity as the N100m component, reaching its maximal strength at around 400 ms and lasting until the end of stimulation.

In children, the small P50m component at around 40 ms was followed by a second deflection of the same positive polarity at 80–84 ms (Fig. 2b, c), which corresponds to the child P100m and is distinct from the P50m and N100m components in adults [37]. The absence of the N100m and the P200m peaks in auditory responses to clicks and tones is typical for children before adolescence [37, 38, 40].

The qualitative difference in the pattern of the transient auditory components between children and adults was confirmed by the presence of ‘positivity’ in children and ‘negativity’ in adults in the ~ 70–90 ms interval, both of which were significant in the right ROI between 74 and 88 ms after stimulation onset (FDR corrected *p* < 0.01 in both children and adults).

Despite the striking developmental difference in morphology of the transient components, the SF in children resembled that in adults: in both age groups, its sources in the auditory cortex had the same direction of current and comparable magnitude (Fig. 2c). Moreover, in response to contralateral stimulation, the SF dominated in the right hemispheres in both age groups. The visibly faster development of the SF in children compared with adults might be explained by the lack of the adult P200m component that masks the early segment of the SF response. In both children and adults, the 40 Hz ASSR overlapping with the SF is clearly visible on sensors (Fig. 2a, b) and at the source level (Fig. 2c).

### Source localization of the 40 Hz ASSR and SF in the auditory cortex

To compare cortical localizations of the ASSR and SF, for each subject we calculated the MNI coordinates of the sources with the maximal 40 Hz ITPC in the 200–500 ms range and those with the maximal integrated SF amplitude in the 200–500 ms range in response to contralateral stimulation. Given that localization results are highly sensitive to SNR, we excluded from the coordinate analysis those subjects who had very low ITPC values or visually undetectable SF in the hemisphere contralateral to the stimulated ear. For ASSR coordinate analysis we excluded those subjects who had ITPC equal to or lower than 0.18, the highest ITPC value observed during the baseline (see the Additional file 7: Figure S2). As a result, 31 NT/33 ASD and 33 NT/32 ASD children were included in the analysis of the ASSR MNI coordinates for the left and right hemispheres, respectively. For the SF coordinate analysis, we excluded subjects/hemispheres where the sustained negative deflection of the field with a rising front slope after the click onset was not detectable (see example in the Additional file 7: Figure S3). This resulted in the inclusion of 33 NT/29 ASD and 35 NT/35 ASD children in the analysis of the SF MNI coordinates for the left and right hemispheres, respectively. Both sets of data that fulfilled the described criteria were available for all adults for both hemispheres, for 29 NT/28 ASD children for the left hemisphere and 33 NT/32 ASD children for the right hemisphere. Except for the analysis of MNI coordinates, all the other analyses of auditory responses were performed for the full sample of participants.

**Fig. 3:**
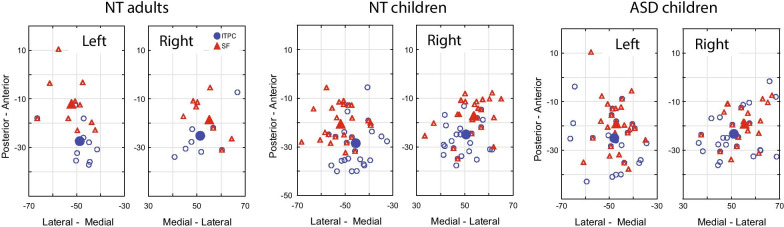
MNI coordinates of the 40 Hz auditory steady-state response (ASSR) inter-trial phase coherence (ITPC, blue shapes) and the sustained field (SF, red shapes) maxima. Localization of the sources in the horizontal plane. Small open circles and triangles correspond to individual coordinates; big filled shapes show the group means

The MNI coordinates of the SF and the 40 Hz ITPC maxima in the three groups of participants are shown in Table 2 and visualized in Fig. 3. In the NT children, in both hemispheres, the SF source was located anterior, lateral and inferior to that of the ASSR (paired *t*-test, left *X*: *t*_(28)_ = 4.8, *p* = 0.00005; left *Y*: *t*_(28)_ = − 3.7, *p* = 0.0008; left *Z*: *t*_(28)_ = 3.1, *p* = 0.004; right *X*: *t*_(32)_ = − 3.4, *p* = 0.002; right *Y*: *t*_(32)_ = − 5.8, *p* = 0.000002; right *Z*: *t*_(32)_ = 3.9, *p* = 0.004). The same relative positions of the ASSR and SF sources in adult participants were previously described by Keceli and colleagues [20] using single dipole modeling. For comparison purposes, Table 2 gives original Talairach and estimated MNI coordinates of the SF and ASSR, reported by Keceli et al. [20]. In our adult sample, the SF maxima were located anterior and inferior to those of the ASSR in both hemispheres (paired *t*-test, left *Y*: *t*_(9)_ = -3.0, *p* = 0.02; left *Z*: *t*_(9)_ = 3.3, *p* = 0.01; right *Y*: *t*_(9)_ = − 2.4, *p* = 0.04; right *Z*: *t*_(9)_ = 1.8, *p* = 0.1), while the lateral shift along the *X* axis was not significant (left *X*: *t*_(9)_ = 1.4, *p* = 0.21; right *X*: *t*_(9)_ = -1.2, *p* = 0.28), possibly because of the small sample size. In children with ASD, the differences in cortical localization between the SF and ASSR sources in the right hemisphere were in the same direction as in the NT children (right *X*: *t*_(30)_ = − 3.0, *p* = 0.005; right *Y*: *t*_(30)_ = − 2.9, *p* = 0.007; right *Z*: *t*_(30)_ = 3.1, *p* = 0.004), while in the left hemisphere the lateral displacement of the SF source relative to the ASSR sources was not significant (left *X*: *t*_(25)_ = − 0.65, *p* = 0.5; left *Y*: *t*_(25)_ = − 3.3, *p* = 0.003; left *Z*: *t*_(25)_ = 1.9, *p* = 0.07).

**Table 2:** Grand average MNI coordinates of the maximal auditory steady-state response (ASSR) and sustained field (SF) sources

Group (N left/N right)	Left hemisphere mean and (SD)			Right hemisphere mean and (SD)		
	*X* (lateral–medial)	*Y* (posterior–anterior)	*Z* (superior–inferior)	*X* (medial–lateral)	Y (posterior–anterior)	*Z* (superior–inferior)
*ASSR*						
NT adults (10/10)	− 48.7 (6.9)	− 27.4 (8.6)	9.7 (6.5)	51.4 (7.8)	− 25.2 (7.7)	10.1 (5.5)
NT children (31/33)	− 45.5 (6.0)	− 28.5 (8.8)	11.0 (6.8)	50.2 (6.0)	− 25.3 (6.6)	11.1 (4.3)
ASD children (30/32)	− 47.8 (8.0)	− 25.0 (9.4)	9.0 (6.8)	51.0 (8.4)	− 23.4 (8.5)	10.4 (4.5)
*Keceli *et al*. 2015*^a^ *A(adults, N = 11)*						
Estimated MNI	− 48	− 22	11	52	− 17	13
Original Talairach	− 45	− 22	13	48.5	16.5	14
*SF*						
NT adults (10/10)	− 52.1 (7.7)	− 12.1 (10.7)	1.2 (6.5)	55.1 (6.8)	− 18.5 (8.3)	6.8 (6.2)
NT children (33/35)	− 51.9 (6.7)	− 20.5 (7.3)	6.6 (3.7)	53.7 (6.6)	− 17.2 (6.6)	7.4 (3.4)
ASD children (29/35)	− 47.1 (6.0)*	− 19.0 (10.4)	5.7 (7.4)	55.5 (6.2)	− 19.1 (6.5)	7.5 (4.3)
*Keceli *et al*. 2015*^a^ *A(adults, N = 12)*						
Estimated MNI	− 51	− 17	7	52	− 13	6
Original Talairach	− 48	− 18	8.5	49	− 14	9

^a^The provided coordinates are approximated from Fig. 3 in [20], where the authors localised the ASSR and SF responses evoked by the same periodic stimuli [20]

^*^Significant difference in ASD versus NT children: *p* = 0.005

For the 40 Hz ITPC maxima in the right or left hemispheres, there were no significant differences between children with and without ASD in either *X*, *Y* or *Z* coordinates (*t*-test, left *X*: *t*_(59)_ = 1.3, *p* = 0.2; left *Y*: *t*_(59)_ = − 1.5, *p* = 0.14; left *Z*: *t*_(59)_ = 1.2, *p* = 0.2; right *X*: *t*_(63)_ = − 0.5, *p* = 0.6; right *Y*: *t*_(63)_ = − 1.1, *p* = 0.3; right *Z*: *t*_(63)_ = 0.6, *p* = 0.5). The SF maximum in the left hemisphere was located significantly more medial in children with ASD than that in NT children, although the difference was relatively small (*X* = coordinate in NT: − 51.9, ASD: − 47.1, *F*_(1,61)_ = 8.5, Cohen’s *d* = 0.74, *p* = 0.005, uncorrected for multiple comparisons). The multivariate Hotelling *T*^2^ test confirmed significant ASD versus NT differences in the source localization (*F*_(3,59)_ = 3.1, *p* = 0.03, partial eta-squared = 0.14). There were no group differences for the SF coordinates in the right hemisphere.

To investigate whether the SF ‘early’ interval (150–250 ms), which was visible only in children, represents the evolving SF, we compared cortical locations of the SF maxima in this interval with those in the 300–500 ms interval, where the sustained field was observed in both children and adults. There were no significant time-related differences in *X*, *Y* or *Z* SF coordinates in the NT, ASD or the combined sample of children in either hemisphere (paired *t*-test, all *p*s > 0.08, incorrected for multiplr comparisons). This means that the onset interval of the SF in children originates from the same region of the auditory cortex as the rest of the SF.

### Comparison of the 40 Hz ASSR in NT children and children with ASD

To analyze group differences in ASSR magnitude, we computed for each participant an average of 40 Hz z-ITPC and power in the common ROI, separately in the right and left hemispheres (see Methods for details). We analyzed group differences in the ASSR parameters (power and z-ITPC) integrated over 200–500 ms interval, as well as group differences in ASSR parameters timecourses over the whole period of the stimulation.

**Fig. 4:**
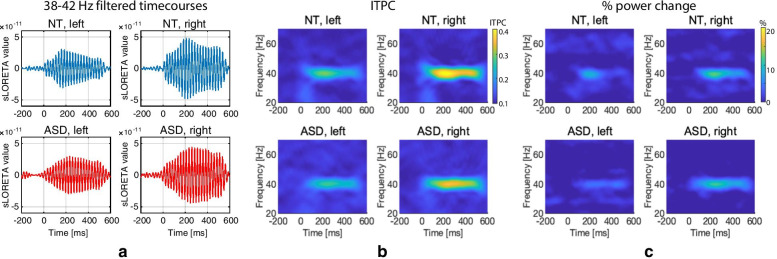
Grand average 40 Hz auditory steady-state response (ASSR) in the right and left hemispheres in neurotypical (NT) children and in children with autism spectrum disorder (ASD). Only responses in the hemisphere contralateral to the stimulated ear are shown. **a** The grand average ASSR waveform filtered between 38 and 42 Hz. (B and C) Time–frequency plots for **b** ASSR inter-trial phase coherence (ITPC) and **c** ASSR spectral power change in percent from baseline (% power change)

Grand averaged ASSR waveforms (filtered between 38 and 42 Hz; FIR filter order = 100) as well ITPC and power time–frequency plots for the left and right common ROIs are presented in Fig. 4. One ASD participant had extremely high ASSR power (the percentage of power change in 200–500 ms interval was more than 5 SDs and 4 SDs above the group mean in the right and left hemispheres, respectively) and was excluded from the analysis (See the Additional file 7: S4 for distribution of the % power change values and Additional file 7: Figure S5 for the time–frequency plots with this subject included).

In both hemispheres, contralateral to the stimulated ear, the integrated z-ITPC values in the common ROI were not normally distributed in NT children (Shapiro–Wilk test, *N*_NT_ = 35, left hemisphere: *W* = 0.94, *p* = 0.07; right hemisphere: *W* = 0.93, *p* = 0.02) and children with ASD (Shapiro–Wilk test, *N*_ASD_ = 34, left hemisphere: *W* = 0.87, *p* < 0.007; right hemisphere: *W* = 0.89, *p* = 0.002). Therefore, we used nonparametric statistical analysis. In both ASD and NT participants, the z-ITPC was greater in the right hemisphere than in the left one (Wilcoxon matched pairs test: *N*_NT_ = 35, *T* = 57, *Z* = 4.2, *p* = 0.00002; *N*_ASD_ = 34, *T* = 226, *Z* = 3.1, *p* = 0.002). There were no group differences in the z-ITPC for either hemisphere (Mann–Whitney *U* test, *N*_NT_ = 35, *N*_ASD_ = 34; left hemisphere: ASD median = 0.63, NT median = 0.77, *U* = 526, *p* = 0.4; right hemisphere: ASD median = 0.95, NT median = 1.57, *U* = 477, *p* = 0.16). Inspection of the distributions of the z-ITPC values corrected for age showed a large overlap between the NT and ASD groups (Additional file 7: Figure S6). The findings for the integrated ASSR power in the common ROI mirrored those for z-ITPC and are given in the Additional file 7: Results.

We repeated the analysis for 30 ‘maximal vertexes’, selected individually for each subject. In this case, the z-ITPC distributions also deviated from normal in NT children (Shapiro–Wilk test, *N*_NT_ = 35, left hemisphere: *W* = 0.92, *p* = 0.02; right hemisphere: *W* = 0.93, *p* = 0.04) and in children with ASD (Shapiro–Wilk test, N_ASD_ = 34, left hemisphere: W = 0.87, *p* = 0.001; right hemisphere: *W* = 0.88, *p* = 0.001). In both ASD and NT participants, the z-ITPC was greater in the right than in the left hemisphere (Wilcoxon matched pairs test: *N*_NT_ = 35, *T* = 71, *Z* = 4.0, *p* = 0.00006; ASD: *N*_ASD_ = 34, *T* = 140, *Z* = 2.7, *p* = 0.007). There were no group differences in the z-ITPC in the individual maxima (Mann–Whitney test, *N*_NT_ = 35, *N*_ASD_ = 34; left hemisphere: ASD median = 0.91, NT median = 0.95, *U* = 521, *p* = 0.38; right hemisphere: ASD median = 1.33, NT median = 1.86, *U* = 451, *p* = 0.09). As with the common ROI, the analysis of the integrated ASSR power in the individual maxima did not reveal any significant group differences (see Additional file 7: Results).

**Fig. 5:**
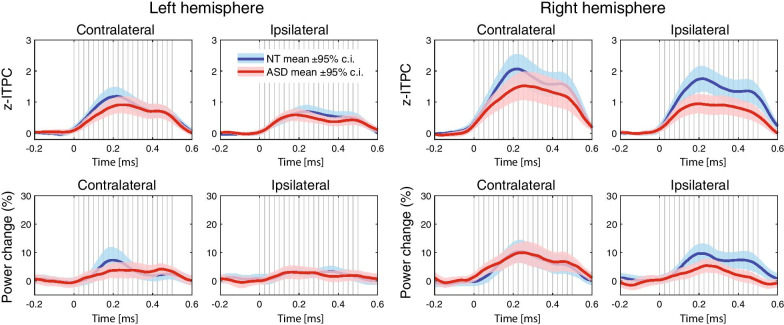
Timecourses of the auditory steady-state response (ASSR) parameters in the left and right hemispheres contralateral and ipsilateral to the monaurally stimulated ear: inter-trial phase coherence (ITPC, upper plots) and power (lower plots) in the neurotypical (NT, blue line) and autism spectrum disorder (ASD, red line) groups. Vertical gray lines mark click onsets. Color-shaded areas denote the 95% two-sided confidence intervals for the respective means; other designations are the same as in Fig. 4. There were no significant group differences (Wilcoxon rank sum test; all false discovery rate corrected *p*s > 0.05)

Figure 5 shows grand average timecourses of the z-ITPC and ASSR power in NT and ASD participants in the left and right hemispheres contralateral and ipsilateral to the stimulated ear. The ASD participant with an extremely high ASSR power (see above) was excluded from this analysis (see the Additional file 7: Figure S7 for the plot with this subject included). There were no statistically significant group differences in any of the ASSR timecourses at any time point (Wilcoxon matched pairs test, all FDR corrected *p*s > 0.05).

**Fig. 6:**
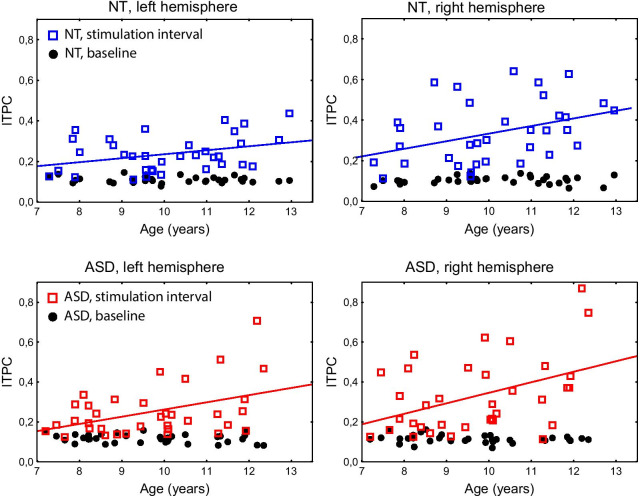
Associations between age and 40 Hz auditory steady-state response (ASSR) in neurotypical (NT) children (upper panels) and children with autism spectrum disorder (ASD, lower panels). The individual stimulus-locked and baseline 40 Hz inter-trial phase coherence (ITPC) values are shown as red/blue squares and black circles, respectively

Figure 6 shows individual 40 Hz ITPC values in children with and without ASD as a function of age. The linear trends suggesting the age-related increase of the 40 Hz ITPC were present in both groups and in both hemispheres, contralateral to the stimulated ear. A developmental increase in the 40 Hz ITPC was confirmed by analysis of the z-ITPC values (Spearman correlation coefficients: *N*_ASD_ = 35, left hemisphere: *R* = 0.30, *p* = 0.08; right hemisphere: *R* = 0.38, *p* = 0.02; *N*_NT_ = 35, left hemisphere: *R* = 0.31, *p* = 0.08; right hemisphere: *R* = 0.34, *p* = 0.048). However, even at younger ages (< 9 years), the majority of children had 40 Hz ITPC values above their baseline level.

To investigate whether the 40 Hz z-ITPC in children with ASD correlated with their intelligence level and severity of autism, we calculated Spearman correlations. Children with more severe autism had *higher* 40 Hz z-ITPC values in the right hemisphere (Table 3). We repeated the analysis for the z-ITPC values after subtracting the linear age trend. This procedure increased the reliability of this result (Spearman *R* = 0.46, *p* = 0.007).

**Table 3:** Spearman correlation between the 40 Hz auditory steady-state response (ASSR) inter-trial phase coherence (ITPC) and psychometric variables in children with autism spectrum disorder (ASD)

	MPI IQ (*N* = 32)	Autism score (*N* = 34)
Left hemisphere	− 0.15, *p* = 0.41	0.16, *p* = 0.35
Right hemisphere	− 0.17, *p* = 0.33	0.38, *p* = 0.026*

^*^Uncorrected for multiple comparisons

### Comparison of the SF in children with and without ASD

**Fig. 7:**
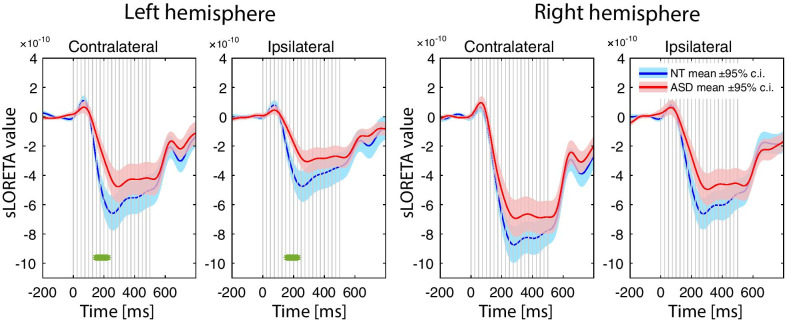
Comparison of the sustained field (SF) responses in the left and right cortical maxima in children with and without autism spectrum disorder (ASD). The signal was low-pass filtered at 9 Hz. Vertical gray lines mark click onsets. The green asterisks under the curves denote significant between-group differences on a point-by-point basis (Wilcoxon rank sum test, *p* < 0.01, false discovery rate correction for multiple comparisons)

As in case of the ASSR, between-group comparisons for the SF timecourses were performed in the source space (see Methods for details). Figure 7 shows the grand average low-passed SF source waveforms in the left and the right hemispheres for the contra- and ipsilateral ear stimulation in the ASD and the NT groups. Visually detectable SF source waveforms were present in the right hemisphere in all participants and in the left hemisphere in the majority of children from both samples, with the exception of two NT and six ASD children.

First, we analyzed the effect of age on the SF maximal amplitude using Pearson correlations. None of the correlations were significant in the NT, ASD or in the combined sample (all *p*s > 0.2; see Additional file 7: Table S1) suggesting that the SF amplitude did not change between 7 and 12 years of age.

**Fig. 8:**
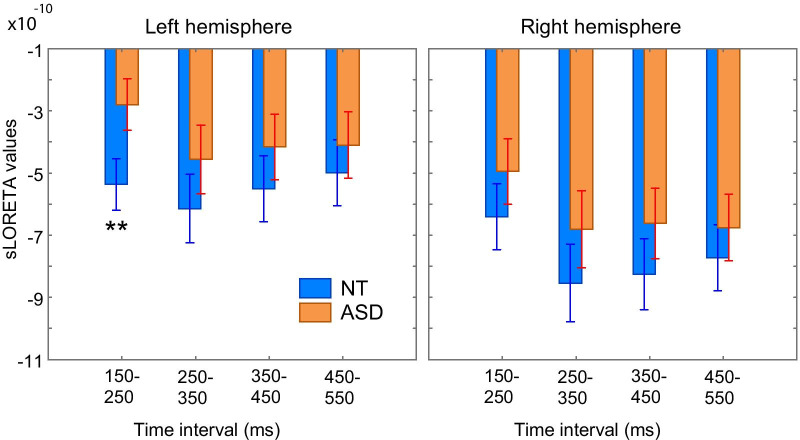
Group differences in the sustained field (SF) source amplitude in the consecutive temporal intervals after the click train onset. The SF responses in the left and the right hemisphere are evoked by stimulation of the contralateral ear. **p < 0.001, Bonferroni corrected

We then examined the effects of hemisphere and contralaterality of the stimulation on the SF maximal amplitude in the NT and ASD groups. To this end, we used rmANOVA with group (ASD, NT), hemisphere (left, right) and ear of stimulation (contralateral, ipsilateral) as the factors and the maximal amplitude of the SF source current in the 150–500 ms stimulation interval as a dependent variable. There were strong effects of hemisphere (*F*_(1,68)_ = 50.5, *p* < 0.00001, partial eta-squared = 0.43) and ear of stimulation (*F*_(1,68)_ = 128.4, *p* < 0.00001, partial eta-squared = 0.65). The SF maximal amplitude was higher in response to the contra- than ipsilateral stimulation, and it was generally higher in the right than in the left hemisphere (Figs. 7, 8). Neither group × hemisphere, nor group × ear interaction effects were significant (both uncorrected *p*s > 0.1), suggesting that the auditory SF response in both groups was characterized by contralaterality and right hemisphere dominance. The SF maximal amplitude was higher in the NT compared with the ASD group (group main effect: *F*_(1,68)_ = 4.6, *p* = 0.035, partial eta-squared = 0.06). This suggests that the SF in children with ASD was reduced compared with NT controls in both hemispheres and in response to both contra- and ipsilateral ear stimulation. Analysis of the point-by-point group differences in the SF timecourses (Wilcoxon matched pairs test, *p* < 0.01, FDR corrected for multiple comparisons) revealed that the SF in children with ASD was particularly strongly attenuated in the left hemisphere approximately between 150 and 220 ms after the click train onset, suggesting an atypically slow rise of the SF source strength in children with ASD (Fig. 7).

For the further analysis of between-group difference in the SF source timecourse, we focused on the contralateral responses that were greater and more reliable than the ipsilateral ones (Fig. 7). To test for the group differences in the SF timecourses, we divided the SF time window (150–550 ms) into four successive time intervals in respect to the click train onset (151–250, 251–350, 351–450 and 451–550 ms), with the first (151–250 ms) interval corresponding to the raising part of the SF. We then performed rmANOVA with time, hemisphere and group as the factors and the average SF source current amplitudes in the 100 ms time intervals as a dependent variable (Table 4, Fig. 7).

**Table 4:** Group differences in the SF source timecourses: repeated measures ANOVA results

	*F*	*p*	G–G epsilon	Partial eta-squared
Group	**5.9**	**0.018**		0.08
Time	**32.4**	**< 1e−6**	0.57	0.32
Time × GR	**3.3**	**0.04**	0.57	0.05
Hemisphere	**43.4**	**< 1e−6**		0.39
Hem × GR	0.04	0.84		0.00
Time × Hem	**9.2**	**0.00001**	0.71	0.12
Time × Hem × GR	**3.7**	**0.024**	0.71	0.05

Children with ASD had generally lower SF source amplitude than NT children, especially during the rising part of the SF timecourse—from 150 to 250 ms after stimulation onset (Fig. 8; time × group interaction: *F*_(3,204)_ = 3.3, adjusted *p* = 0.04). The significant time × hemisphere × group interaction (*F*_(3,204)_ = 3.7, adjusted *p* = 0.024) was due to a greater and more reliable reduction of the ‘early’ SF segment in the left hemisphere than in the right one in children with ASD (ASD vs NT left hemisphere: Bonferroni-corrected *p* < 0.001; right hemisphere: n.s.). Except for the ‘early’ SF amplitude in the left hemisphere, none of the between-group differences survived after Bonferroni correction for multiple comparisons. This result is consistent with the data presented in Fig. 7. The effect size (Cohen’s d = 1.01) for the difference between ASD and NT groups in the SF_150–250_ amplitude was classified as a large effect based on benchmarks suggested by Cohen (in [61]). To check whether the group differences could be explained by a delayed maturation of the SF in ASD children, we calculated Pearson correlations between age and the averaged SF source amplitudes in the four time intervals after the stimulation onset in the left and right auditory cortices. There were no significant correlations with age for the SF source amplitudes in either the NT, ASD or combined groups (all uncorrected *p*s > 0.2; see Additional file 7: Table S1).

To check if the timing of the observed group differences in the SF amplitude was affected by application of the low-pass filter to the averaged waveform, we repeated the analysis presented in Fig. 7 and Table 4 using unfiltered signal. The results remained principally the same (see the Additional file 7: Figure S8 and Table S2).

To test whether the group differences in the SF were due to lower IQ in ASD children, we repeated the ANOVA for the subsample of 10 ASD and 10 NT participants who were matched both for IQ level (MPI standard score: NT = 109.7 ± 10.2; ASD = 109.4 ± 11.9) and age (age in years, mean ± SD: NT = 10.0 ± 1.4, ASD = 9.2 ± 1.5). Due to a small number of participants, we used the non-parametric Mann–Whitney *U* test for between-group comparisons. The significant difference between ASD vs NT groups was again detected only in the early time interval in the left hemisphere (SF_150-250_, Left hemisphere, ASD median = − 2.55 (a.u.), NT median = − 4.94 (a.u.), *U* = 16, *Z* = − 2.53; *p* = 0.01; see Additional file 7: Figure S9). The results suggest that the slowing of neural activation in the left auditory cortex evoked by click trains characterized ASD children irrespective of the presence of cognitive difficulties.

Considering that there were small, but significant group differences in the SF localization in the left hemisphere (Table 2), we wanted to ensure that the between-group differences in the SF source amplitude were not driven by the choice of the SF vertices for the group analysis, which was done by averaging across the NT and ASD data (see Methods for details). For this purpose, we repeated the rmANOVA analysis for the SF source amplitude calculated in the individually chosen 30 vertices with maximal SF amplitudes. The results remained principally the same (GR: *F*_(1,68)_ = 3.7, *p* = 0.057, partial eta-squared = 0.05; time × group: *F*_(3,204)_ = 3.2, adjusted *p* = 0.047, partial eta-squared = 0.05; time × hemisphere × group: *F*_(3,204)_ = 5.6, adjusted *p* = 0.003, partial eta-squared = 0.08). The left-hemispheric reduction of the SF amplitude in the 150–250 ms range remained significant: *F*_(1,68)_ = 11.2, Bonferroni corrected *p* < 0.05.

To summarize, in both the NT and ASD groups, the SF source strength was higher contralaterally to the stimulated ear and clearly dominated in the right hemisphere. In the ASD group, the SF was moderately reduced in both hemispheres and strongly delayed in the left one, irrespective of the stimulated ear. None of the SF parameters significantly changed with age between 7 and 12 years in either the NT or ASD group.

We performed additional correlation analysis to investigate whether the two principle findings—the bilateral reduction of the SF maximal amplitude and the left-hemispheric SF reduction in its 150–250 ms early interval (‘SF delay’) in children with ASD—are associated with psychometric variables (Table 5). Because children with lower MPI IQ scores had more severe autism (Pearson *R* = − 0.36, *p* = 0.049), to estimate independent associations of these psychometric variables with the SF amplitude, we calculated partial correlations. We performed this analysis in 31 children with ASD in whom both MPI IQ and Autism Scores were available (Table 5). Although the maximal SF source amplitude (SF_max_) was decreased (less negative) in children with ASD at the group level, its greater strength in the right hemisphere correlated with greater severity of autism traits (*R* = − 0.64, Bonferroni-corrected *p* < 0.001). The SF_max_ amplitude in both hemispheres also tended to be higher in the ASD participants with higher IQ, but these correlations did not survive after Bonferroni correction. Thus, the results of the correlation analysis mainly indicate that the higher SF amplitude in the right hemisphere in children with ASD is associated with greater severity of their autism symptoms.

**Table 5:** Partial correlations between the sustained field (SF) source current* and psychometric variables in children with ASD (N = 31)

SF amplitude	MPI IQ: r, p**	Autism Score
SF_max_, Left hemisphere	− 0.35*, *p* = 0.06	− 0.22, *p* = 0.25
SF_max_, Right hemisphere	− 0.43, *p* = 0.017	− **0.64, p = 0.0001**
SF_150–250_, Left hemisphere	− 0.06, *p* = 0.74	− 0.04, *p* = 0.85
SF_150–250_, Right hemisphere	− 0.14, *p* = 0.47	− 0.41, *p* = 0.026

^*^Note that the SF current is negative, and a negative correlation reflects a direct link between the SF strength and a respective psychometric variable

^**^Uncorrected for multiple comparisons; *p* value that remained significant after Bonferroni correction is shown in bold type

## Discussion

We investigated putative hemispheric differences in low-level cortical processing of periodic spectrally complex sound in boys with ASD and age-matched control boys. To this end, we used 40 Hz click trains presented separately for the left and right ears to probe contralateral auditory responses. In children, similar to adults, temporally regular clicks evoked two types of sustained neural responses with different sources in Heschl’s gyrus: the ASSR and SF. Regardless of age, the SF sources in both hemispheres were positioned anterolateral to those of the ASSR. These results agree with the previous report that showed similar relative positions of neural generators of these components in adults [20]. While the ASSR in children with ASD did not distinguish them from their typically developing peers, their SF was reduced in both hemispheres, as well as markedly delayed and spatially displaced in the left hemisphere. Below, we will discuss the implications of these results in the context of previous neuroimaging and neuronal studies of temporal regularity processing in the auditory cortex.

### ASSR in NT children and children with ASD

In accordance with the previous findings in adults [21, 62], monaural stimulation in children elicited the ASSR with significant contralateral predominance and generally higher magnitude in the right hemisphere (Figs. 4, 5). In both children and adults, the ASSR was localized in or in vicinity of the primary auditory cortex (area A1) (Fig. 3). Despite differences in source localization techniques, the MNI coordinates of the ASSR maxima in our study were similar to those previously reported by studies performed in adults using single dipoles anatomically constrained to the left and right Heschl’s gyri [20, 62].

The phase consistency of the ASSR—the most individually reliable feature of this type of response to periodic clicks [54]—increased between 7 and 12 years of age (Fig. 6). This finding is in line with the available evidence of marked enhancement in the 40-Hz ASSR between late childhood (8–10 years) and early adolescence [29, 63–65]. This age-related increase can be explained by developmental changes in fast-spiking parvalbumin-sensitive neurons involved in the generation of sensory gamma oscillations and their entrainment by periodic auditory stimuli [63].

In the majority of children in our study, the 40 Hz ITPC values were clearly above the baseline (Fig. 6). This result contrasts with that of Edgar et al. [29], who found that the majority of 48 NT children aged 7–14 years lacked a discernible 40 Hz ITPC responses. The higher proportion of children with reliable ASSR in our study can be explained by methodological differences, such as the use of clicks instead of tones, monaural instead of binaural stimulation, source localization methods, etc. Whatever the reason, our paradigm allowed us to detect ASSRs in most children. This is important, because Edgar et al. suggested that inaccurate ASSR measurement during childhood might preclude the observation of atypical ASSR in the pediatric populations with neuropsychiatric disorders, such as ASD.

Despite the improved SNR, similarly to Edgar et al. [29] and Ono et al. [22], we found no significant difference in ASSR between the ASD and NT groups of children (Fig. 4). Also in accordance with the Edgar et al. study, children with ASD demonstrated a normal developmental increase in ASSR in both hemispheres (Fig. 6), which strengthens the conclusion that the ASSR matures normally between 7 and 12 years of age in children with ASD, at least at the group level. Our findings and those of Edgar et al. differ from the results of Wilson et al. [25], who observed a reduced left-hemispheric 40 Hz ASSR to monaural clicks in 10 children with ASD compared with 10 NT children. We should note, however, that the small sample size in this frequently cited research reduces the likelihood that a statistically significant result reflects the true effect [66]. Overall, our results demonstrate fairly typical development of the ASSR in the primary auditory cortex in children with ASD, at least until puberty. In this context, the reduction of the ASSR found in adolescents and adults with ASD [24, 30] or parents of children with ASD [28], but not in children with ASD ([22, 29, 30], but see [25]) may indicate that the maturation of this auditory response in ASD diverges from the normal trajectory during or after puberty.

It is noteworthy that some children with ASD had z-ITPC values that were very high for their age (Additional file 7: Figure S6). Moreover, the magnitude of the 40-Hz ITPC in the contralateral right hemisphere was *higher* in those children with ASD who had more severe behavioral symptoms of autism (Table 3). Although, given the results of previous ASD studies, this was an unexpected finding, it is consistent with a direct correlation between the magnitude of the 40 Hz ASSR and the severity of positive symptoms in patients with schizophrenia [67]. It is likely that one of the factors that increases ASSR is the high excitability of the auditory cortex. It has been suggested that an increased propensity for high-frequency synchronisation in the primary auditory cortex indicates its hyper-excitable state [68]. This hypothesis is compatible with our findings as well as with the animal data on augmented ASSR under high arousal states [68].

Although our findings do not support the idea of the atypically reduced (or enhanced) ASSR as a common feature among children with ASD, the typical ASSR may not be necessarily representative of all children with this diagnosis. Taking into account the large genetic heterogeneity of ASD [69], the ASSR may be altered in a certain sub-group of ASD population. Further ASSR research targeting the specific ASD-associated molecular-genetic pathways that may affect the ASSR (see e.g. [70]), would be valuable.

### SF in NT children and adults and in children with ASD

We found that in children, similarly to adults [18], the sustained neural response to temporally regular 40 Hz clicks consists of two superimposed neural signals: the ASSR and slowly developing negative DC shift, referred to as the SF. The current of the SF source has the same direction in both age groups (Fig. 2), which confirms functional homology of the SF in children and adults. It has been suggested that in adults the SF onset is hidden because it overlaps with the sequence of obligatory transient responses to the stimulation onset: P50m, N100m and P200m [18]. The same is probably true in children. The seemingly earlier onset of SF in children than in adults might be explained by the absence of obligatory components with longer latencies (N100m and P200m) in the immature auditory evoked field (Fig. 2, see also [37–39]). Apart from difference in the onset time, the properties of SF in children are similar to those in adults.

*First*, distributed localization modeling shows that in both children and adults, SF maxima are located in or in the vicinity of the core of the auditory cortex, contralateral to the stimulated ear and anterolateral to the ASSR maxima (Fig. 3; Table 3). The results of MEG inverse solutions depend on many factors, including the forward models used [71]. Therefore, the exact MEG-derived coordinates of the SF and ASSR sources should be treated with caution. Nevertheless, because the *same relative position* of the ASSR and SF sources has been reported by Keceli et al., who used a localization technique different from ours—single dipole source modeling [20]—we are confident in the validity of these results. Besides, the group average coordinates of the SF maxima in our participants are remarkably similar to the coordinates of the ‘anterior SF source’ derived from a four-dipole model in the study of Gutschalk et al. (see Table 1 in [18]). It is therefore likely that, similarly to adults, the SF in children is generated in the pitch processing centre [19, 34, 62] located immediately anterolateral to the primary auditory cortex in the lateral Heschl’s gyrus [12, 16].

*Second*, the dependence of the SF strength on the stimulated ear is identical in children and adults—in both groups the SF has greater amplitude in the hemisphere contralateral to the stimulated ear, and, in general, in the right hemisphere (Fig. 2). The same hemispheric asymmetry had been described for adults [21].

Unlike the ASSR, the SF in children with ASD was clearly different from that in the NT children. This dissociation between the two complementary neural responses to periodical clicks provides a clue to the lowest level of the cortical hierarchy at which the periodicity processing may be impaired in children with ASD.

The temporal regularity at a frequency of 40 Hz is explicitly represented in monkeys’ area A1 by neuronal firing patterns synchronised with each individual click [72]. The human ASSR, most probably, reflects this ‘stimulus-synchronised code’ implemented in area A1 [73]. However, the ASSR is thought to reflect neural coding of relatively slowly repeating acoustic events (up to 50 Hz) [73]. The tonotopically organised A1 neurons, although involved in coding pure tones over a wide range of frequencies, are limited in their capacity to synchronise with the periodic modulations in spectrally complex sounds at rates faster than about 100 Hz [72].

The click repetition rate of 40 Hz, which is above the lower frequency limit for perceiving the click train as a continuous sound (approximately 30 Hz; [8]), also activates the second neural code for temporal regularity—the monotonous rate-coding [9, 11, 72]. Perception of pitch in harmonic continuous sounds corresponds to the lowest rate at which the periodic waveform repeats itself, and the inverse of this period is called the *f*_*0*_ [45]. It has been shown that in monkeys, increasing the *f*_*0*_ of the click train is reflected in a monotonous increase in the firing rate of neurons located in the cortical field rostral to the A1, which is homologous to the human pitch-processing center in the auditory core area [9]. Given that the neuronal firing rate in cortical evoked responses is associated with surface-negative DC shift in non-invasive EEG/MEG (for a review, see [74]), it is conceivable that the SF reflects activity of the rate-coding neurons in the pitch-processing center [62]. Indeed, similarly to the neuronal firing rate in monkeys, the anterior SF power in humans is directly proportional to the *f*_*0*_ of a click train and is strongly sensitive to violations of temporal periodicity, both in click trains and in human vowels [34]. Gustchalk et al. argued that the anterolateral portion of Heschl’s gyrus—the area that generates the anterior SF—serves to integrate pitch information across different frequency channels and/or calculates the specific pitch value in spectrally complex sounds.

Based on these considerations, the coexistence of normal ASSR (Figs. 4, 5) with reduced and delayed SF (Figs. 7, 8) in our participants with ASD is consistent with the emerging picture of dual temporal encoding of 40 Hz fundamental frequency in the human auditory core [9, 73]. While the ‘stimulus-synchronized code’ employed by the primary auditory cortex seems to be relatively spared in children with ASD, the ‘rate-coding’ implemented in the anterolateral region of Heschl’s gyrus might be disrupted.

The undisturbed functioning of the primary auditory cortex in children with ASD is well in line with numerous electrophysiological evidence on their typical, or even enhanced processing of frequency/pitch of pure tones (for a review, see [75–77]) that mainly relies upon A1 neural circuitry. On the contrary, the SF delay suggestive of abnormal functioning of the ‘pitch processing center’ in our participants with ASD generally agree with the conclusion derived from the comprehensive meta-analysis of mismatch-negativity (MMN) studies in autism [77]. The results of this meta-analysis point to weak neural encoding of *spectrally and/or temporally complex non-speech stimuli* in prepubertal children with ASD. However, the previous MMN research clarified neither the cortical level at which this deficit occurs nor its hemispheric lateralization in children with ASD.

In this respect, the most important and intriguing finding of the current study is the left-hemispheric preponderance of the SF delay in children with ASD. The SF was markedly delayed in the left hemisphere, while its maximal strength was slightly, but significantly, reduced in both hemispheres in children with ASD (Figs. 7, 8). Moreover, the source of the SF maximum in the left hemisphere in children with ASD was located more medially than that in the control group.

There are several potential factors that could obscure the measurements of auditory evoked fields, but they can hardly explain their SF delay in children with ASD found in our study. Poor-quality MEG recording in children with ASD (see [77] for a discussion) is unlikely to explain the group differences. Indeed, the ASSR phase consistency did not differ between the ASD and NT groups (Fig. 4), pointing to comparable SNR. Hampered involuntary deployment of attention to salient auditory stimulation, such as phonemes in children with ASD (see [78, 79] for a discussion), can also be refuted due to a neutral, purely sensory nature of the stimulation in our study. Besides, differences in attentiveness to the flow of auditory stimulation would equally affect AEFs in both hemispheres and cannot explain the observed left-hemispheric SF deficit in children with ASD. Differences in automatic orienting to an abrupt onset of click stimulation in ASD [37] affect the auditory responses predominantly in the right hemisphere [80–82] and could not elicit selective left-hemispheric delay of SF in our ASD participants. The delayed maturation of the N100m in the clinical group could affect the activation strength around 150–200 ms after the click train onset, because the N100m has the same direction of current as the SF and, if present, partially overlaps with its early segment. However, the N100m matures earlier in the right than in the left hemisphere [37, 38], and its delayed maturation would rather affect the right-hemispheric SF in children with ASD. The lack of significant correlations between amplitude of the SF in its ‘early’ segment (150–250 ms) and age makes this explanation even more unlikely. To sum up, the atypically slow evolving SF in the left hemisphere in children with ASD appears to be a true phenomenon and is not a result of the ‘developmental delay’ or a methodological artifact.

Remarkably, we observed a small but significant group difference in the source location of the SF in the left but not in the right hemisphere. In previous studies in people with ASD, researchers have reported morphological abnormalities in language-related areas of the left temporal lobe [83–85], but also in Heschl’s gyri [86]. We did not perform structural brain analysis, and it is therefore unclear whether the left-hemispheric difference in SF localization reflects primarily functional or morphological differences between the groups. Nevertheless, this finding is generally in line with our hypothesis regarding the dysfunction of the left-hemispheric pitch-processing center in ASD.

The functional relevance of the left-hemisphere SF delay in children with ASD can be explained in light of the different functional roles of the left and right anterolateral regions of the auditory core in processing the fundamental frequency/pitch of spectrally complex harmonic sounds. Periodicity in complex harmonic sounds is analyzed in the auditory system through two mechanisms that play different but complementary roles (for a review, see [9]). One of them performs rapid extraction of the *f*_0_—i.e. the repetition rate of the temporal envelope—while another relies upon spectral calculation of the relationships between harmonics (*f*_sp_). The latter mode is more precise and universal because it works even if the *f*_*0*_ is not physically present in the stimulus. The 40 Hz click trains used in our experiment were characterized by the presence of both *f*_0_ and its higher frequency harmonics (Fig. 1), which implies the involvement of both mechanisms in the processing of this signal. Although it is generally accepted that pitch processing in music and speech is lateralized to the right hemisphere (e.g. [87]), a number of neuroimaging and lesion studies suggest a stronger sensitivity of the right hemisphere to spectral properties of periodic sounds (for a review, see [88]), and a relative left-hemispheric specialization for rapid decoding of its fundamental frequency [89, 90]. In particular, a larger grey matter volume of the left anterolateral Heschl’s gyrus predisposes people to hear the *f*_*0*_ in the ambiguous sound, while a greater volume of this region in the right hemisphere inclines them to rely on the *f*_sp_ when processing pitch [16].

There is strong evidence that such low-level left-hemispheric specialization for processing of the temporal envelope is directly associated with the capacity to incorporate pitch information into perception of speech [17]. Wong et al. reported that people who were more successful at learning how to use pitch difference to discriminate unknown pseudowords and associate them with visual images also had greater grey matter volume in the left Heschl’s gyrus (but not in the right Heschl’s gyrus).

Given the above, we suggest that selective slowing of neural activation in the left anterolateral Heschl’s gyrus in children with ASD may hinder their linguistic pitch processing, even if their perception of musical pitch is preserved. Although speculative, this explanation is consistent with the paradoxical dissociation between an undisturbed or even enhanced ability to perceive melodic contour (pitch changes) in non-speech stimuli in people with ASD [91, 92] and their difficulties with decoding and producing pitch variations (e.g. intonation) in speech [93, 94]. Interestingly, this dissociation is present even in tone language speakers with autism, suggesting that the tone language experience does not compensate for their speech intonation perception deficit [95]. The difficulties with linguistic pitch processing and production in people with ASD are relatively independent from their intelligence, severity of communication disturbances and even from the other language skills [93, 96]. This may explain the absence of correlation between the ‘early’ SF and psychometric variables in our study (Table 5).

At this point, it is unclear whether the lateralized SF delay observed in children with ASD in our study is specific for processing of the temporal regularities in sound or reflect generally disturbed left hemisphere specialization across multiple cortical areas that has been found in ASD individuals in structural MRI studies [97, 98].

At first glance, our conclusion about predominantly left-hemispheric delay of the neuromagnetic auditory response to clicks in children with ASD disagrees with the previous reports about bilateral or even exclusively right-hemispheric prolongation of the M100 latency in response to pure tones in individuals with autism [99–101]. However, we do not believe that our results contradict these findings. The lateralization of event-related field (ERF) abnormalities in children with ASD may depend on the type of the auditory stimulation (click trains vs pure tones). The SF evoked by click trains may reflect processing of periodicity of a spectrally complex sound at the level of the ‘pitch processing center’, while responses to the pure tones are encoded in the primary visual cortex by neurons, which provide tonotopic representation of sound frequency [11, 45]. Moreover, because M100 and SF components have different developmental trajectories, the group differences in the ERF hemispheric asymmetry may be affected by age. Consistent with the late maturation of the ‘negative’ M100 ([37, 99]; see also Fig. 2), its right hemispheric delay was found primarily in older children with ASD (11–14 years) [99], whereas the left-hemispheric slowing of the SF response in children with ASD in our present study was found at younger age (7–12 years; Figs. 7, 8).

In contrast to the left-hemispheric delay in the SF amplitude growth, its maximal amplitude was slightly, but significantly, reduced in both hemispheres in children with ASD (Figs. 7, 8). Unexpectedly, in the right hemisphere both greater SF strength and higher 40 Hz ITPC were associated with *greater* severity of behavioral symptoms of autism (Tables 3, 5). Both these correlations can be explained by inter-individual variations in a certain factor, such as increased neural excitability of the right auditory cortex, that non-specifically affects the auditory evoked responses in some children with ASD.

To summarize, the monaural presentation of the periodic 40 Hz click trains evokes an unremarkable ASSR but an atypically delayed SF response in children with ASD. The SF, which was generated in Heschl’s gyrus, anterolateral to the ASSR source, was bilaterally reduced and strongly delayed in the left hemisphere contralateral to the stimulated ear in children with ASD. With regard to function, the anterolateral part of Heschl’s gyrus plays a role in processing the pitch of harmonic complex sounds, including speech. The atypically slow build-up of the SF response associated with pitch processing was present in children with ASD irrespective of their IQ and severity of autism symptoms. In this respect, this neuro-functional deficit resembles the well-known difficulties with perception and production of *f*_0_ in speech intonation, which is also present in people with ASD regardless their IQ and autism severity. We assume that deficient low-level processing of fundamental frequency in the ‘pitch processing center' of the left hemisphere may contribute to the abnormal perception and production of speech prosody in people with ASD.

## Limitations

Our study has several important limitations. First, our findings are confined to 7–12-year-old boys. Given the abundant evidence of dramatic developmental changes in the auditory cortex during puberty, future studies should clarify how both types of auditory neural responses to temporal regularity—the ASSR and SF—change during and after this age and whether their development trajectories in people with ASD begin to deviate from the typical ones after puberty. In addition, it is unclear whether our findings may be generalized to girls with ASD. Second, our stimulation protocol did not directly contrast regular interval noise and aperiodic noise stimuli (see e.g. [19]). Therefore, our statement about sensitivity of the child SF to stimulus periodicity is based on previous MEG studies in adults and needs to be tested experimentally in future studies. Besides, we applied fixed inter-stimulus intervals between the click trains, and although this is unlikely to be the main cause of the differences in neural response to click periodicity between ASD and TD children, further experiments with randomly varying ISI will be required to completely rule out this possibility. Third, because we used only 40 Hz click trains, we cannot completely exclude that the SF left-hemispheric delay in the ASD population is confined to this repetition rate, which is only slightly above the lower limit of pitch perception [8]. Fourth, in the present study we did not test the ability of our participants to process vocal and non-vocal pitch. In future studies we plan to combine the SF measurements with assessment of prosody perception and production in children with ASD.

## Conclusions

Our study provides the first evidence of specific left-hemispheric delay in the sustained neuromagnetic response to 40 Hz click trains in children with ASD. Given that this neural response is associated with processing of sound envelope periodicities pertinent to pitch perception, we suggest that its left-hemispheric deficit may be related to selective difficulties with pitch processing in speech contexts experienced by children with ASD. Our findings appear to be the first to show that impaired specialization of the left auditory cortex in children with ASD is not restricted to the high-level abstract representations of vocal speech. Instead, it occurs already at the low-level processing stage mainly concerned with extracting temporal regularity in a time varying acoustic signal.

## Supplementary information

**Additional file 1.** Supplementary video: activation timecourse in the left hemisphere of NT children.

**Additional file 2.** Supplementary video: activation timecourse in the right hemisphere of NT children.

**Additional file 3.** Supplementary video: activation timecourse in the left hemisphere of ASD children.

**Additional file 4.** Supplementary video: activation timecourse in the right hemisphere of ASD children.

**Additional file 5.** Supplementary video: activation timecourse in the left hemisphere of NT adults.

**Additional file 6.** Supplementary video: activation timecourse in the right hemisphere of NT adults.

**Additional file 7.** Supplementary figures and results.

## Data Availability

The datasets analyzed in the current study is available from the corresponding author on reasonable request.

## Abbreviations

AEF: Auditory evoked field
ASD: Autism spectrum disorders
ASSR: Auditory steady state response
ITPC: Inter-trial phase coherence
MEG: Magnetoencephalogram
NT: Neurotypical
SF: Sustained field
