# Supplementary material for: Left hemispheric deficit in the sustained neuromagnetic response to periodic click trains in children with ASD
Source: Mol Autism. 2020 Dec 31;11:100. doi: 10.1186/s13229-020-00408-4 (PMC7775632; doi:10.1186/s13229-020-00408-4)
Supplement: Supplementary file 7 — Additional file 7. Supplementary figures and results. [file 13229_2020_408_MOESM7_ESM.docx]

**Additional file 7**

**Left hemispheric deficit in the sustained neuromagnetic response to periodic click trains in children with ASD.**

Stroganova T.A.^1^, Komarov K.S^1^, Sysoeva O.V^3^, Goiaeva D.E. ^1^, Obukhova T.S. ^1^, Ovsiannikova T.M. ^1^, Prokofyev A.O. ^1^, Orekhova E.V. ^1,2^*

1 - Center for Neurocognitive Research (MEG Center), Moscow State University of Psychology and Education, Moscow, Russian Federation.

2 – MedTech West and the Institute of Neuroscience and Physiology, Sahlgrenska Academy, the University of Gothenburg, Gothenburg, Sweden.

3 – Institute of Higher Nervous Activity, Russian Academy of Science, Moscow, Russian Federation

***Additional file 7: Figure S1*.** Number of artifact-free trials in NT and ASD children.

***Additional file 7: Figure S2****.* Histogram of the ASSR ITPC values in the baseline (-400 to -100 ms) and stimulation (200 to 500 ms) intervals. The data are combined for the ASD and NT group and for the left and the right hemispheres. The subjects/hemispheres with close to the baseline ITPC values (<0.18), were excluded from analysis of ITPC source coordinates, but were included in all other types of analyses.

***Additional file 7: Figure S3.*** Example of a subject with no detectable SF in the left hemisphere: source current time courses in 30 ‘maximal vertices’ (thin lines) and the grand average (thick yellow line). This subject was excluded from analysis of the SF MNI coordinates in the left hemisphere, but included in the SF coordinate analysis in the right hemisphere.

***Additional file 7: Figure S4.*** Histograms of the ASSR power (% change relative to baseline) in NT and ASD children. The outlier is marked by an asterisk.

***Additional file 7: Figure S5.*** Time frequency plots for the ASSR ITPC and power change in NT children and in children with ASD (all subjects are included: N_NT_=35, N_ASD_=35).

***Additional file 7: Figure S6.*** Histograms of the z-ITPC residuals in NT and ASD children after subtraction of the linear age trend. The outlier is marked by an asterisk.

***Additional file 7: Figure S7.*** Timecourses of the 40 Hz ASSR: z-ITPC (upper plots) and % power change (lower plots) in the NT and ASD groups (all subjects are included: N_NT_=35, N_ASD_=35). No group differences were found (all ps>0.05, FDR corrected).

***Additional file 7: Figure S8.*** Comparison of the sustained field (SF) responses in the left and right cortical maxima in children with and without autism spectrum disorder (ASD). *The low-pass filter was not applied to the average SF waveform.* Vertical gray lines mark click onsets. The green asterisks under the curves denote significant between-group differences on a point-by-point basis (Wilcoxon rank sum test, p < 0.01, false discovery rate correction for multiple comparisons). The group differences were significant at 141-190; 200-221; 226-229 ms time points in the left hemisphere contralateral to the stimulated ear and at 165-217 ms in the ipsilateral left hemisphere. Note, that after application of the 9 Hz low-passed filter (Fig. 7 of the Manuscript) the group differences were significant in approximately the same time range: at 146-226 ms in the left hemisphere contralateral to the stimulated ear and at 162-220 ms in the ipsilateral left hemisphere.

***Additional file 7:* *Figure S9.*** Differences in the SF source amplitude in the consecutive temporal intervals after the click train onset in ten children with ASD and ten NT children matched for IQ (NT: 109.7±10.2; ASD: 109.4 ±11.9) and age (NT: 10.0±1.4; ASD: 9.2 ±1.5). The SF responses in the left and the right hemisphere are evoked by stimulation of the contralateral ear. The data are shown for the hemispheres contralateral to the stimulated ear. *p =0.01, Mann-Whitney U test.

***Additional file 7: Table S1.*** Pearson correlations between age and SF amplitudes in the left (LH) and right (RH) hemispheres contralateral to the monaural stimulation.

| Interval | NT | | ASD | | NT + ASD | |
| --- | --- | --- | --- | --- | --- | --- |
|  | LH | RH | LH | RH | LH | RH |
| 150 – 250 ms | -.12 | -.16 | .01 | .19 | -.13 | -0.02 |
| 250 – 350 ms | -.11 | -.02 | -.04 | 0.21 | -.11 | 0.07 |
| 350 – 450 ms | -.11 | .00 | -0.02 | .14 | -0.09 | 0.05 |
| 450 – 550 ms | -.05 | .13 | 0.01 | .14 | -0.03 | 0.11 |

None of the correlations was significant: all p’s>0.2

***Additional file 7: Table S2.*** Group differences in the SF source timecourses: repeated measures ANOVA results. *The low-pass filter was not applied to the SF averaged waveform.*

|  | *F* | *p* | *G-G epsilon* | *partial*  *eta-squared* |
| --- | --- | --- | --- | --- |
| *Group* | **5.9** | **0.017** |  | 0.08 |
| *Time* | **32.5** | **<1e-6** | 0.58 | 0.32 |
| *Time x GR* | 3.3 | 0.05 | 0.58 | 0.04 |
| *Hemisphere* | **43.0** | **<1e-6** |  | 0.39 |
| *Hem x GR* | 0.04 | 0.84 |  | 0.00 |
| *Time x Hem* | **8.8** | **0.00001** | 0.72 | 0.12 |
| *Time x Hem x GR* | **3.8** | **0.022** | 0.72 | 0.05 |

**Additional file 7: Results: 40 Hz ASSR power analysis in the hemisphere contralateral to the stimulation**

Figure S4 shows distribution of 40 Hz ASSR power values in NT and ASD groups, in the left and right hemispheres contralateral to the stimulation. One ASD subject with extremely high % power changes (>5 SD of the mean in the right hemisphere and > 4 SD of the mean in the left hemisphere has been excluded from statistical analysis of ASSR power.

***Common ROI***

Distributions of the ASSR relative power ([stim-baseline}/baseline *100%) differed from normal in NT children (Shapiro-Wilk test (N_NT_=35), left hemisphere: W=0.84, p=0.0002; right hemisphere: W=0.9, p=0.003) and in children with ASD (Shapiro-Wilk (N_ASD_=34), left hemisphere: W=0.84, p=0.0002; Right hemisphere: W=0.90, p=0.003). Therefore, for statistical analysis we used nonparametric statistics.

The ASSR power was higher in the right hemisphere (Wilcoxon matched pairs test: NT: NNT=35, T=194, Z=2.0, p=0.048, ASD: NASD=34, T=159, Z=2.4, p=0.02). No group differences in ASSR power were found (Mann-Whitney test (N_NT_=35, N_ASD_=34), left hemisphere: NT median =2.13, ASD median = 2.05, U=542, p=0.53 right hemisphere: NT median =3.44, ASD median = 4.41, U=551, p=0.60).

Spearman correlations were calculated to analyze developmental change in the ASSR power. The correlations were not significant (NT: N_NT_=35, left hemisphere: R=0.15, p=0.4, right hemisphere: R=0.22, p= 0.2; ASD: N_ASD_=34, left hemisphere: R= 0.15, p=0.39, right hemisphere: R= 0.22, p= 0.20).

***Individually-based ‘30 maximal vertices’***

Distributions of the ASSR relative power differed from normal in NT children (Shapiro-Wilk test N_NT_=35, left hemisphere: W=0.88, p=0.002; right hemisphere: W=0.9, p=0.004) and in children with ASD (Shapiro-Wilk, N_ASD_=34, left hemisphere: W=0.96, p=0.02; Right hemisphere: W=0.87, p<1e-6). Therefore, for statistical analysis we used nonparametric statistics.

In NT participants the ASSR power tended to be higher in the right hemisphere (Wilcoxon matched pairs test: N_NT_ =35, T=203, Z=1.8, p=0.06), while in ASD no hemispheric difference was found (N_ASD_=34, T=283, Z=0.2, p=0.8). There were no group differences in the ASSR power (Mann-Whitney U test, N_NT_=35, N_ASD_=34, left hemisphere: U=514, p=0.33 right hemisphere: U=559, p=0.67).

Spearman correlations were calculated to analyze developmental change in the ASSR power in the individually selected labels. The correlations were not significant (NT left hemisphere: R=0.28, p=0.09, right hemisphere: R=0.24, p= 0.33; ASD left hemisphere: R= 0.05, p=0.7, right hemisphere: R= 0.21, p= 0.2).
